# An atypical presentation of a severe and massive atheroembolic disease

**DOI:** 10.1590/2175-8239-JBN-2018-0013

**Published:** 2018-08-20

**Authors:** Luis Pedro Falcão, Sara Fernandes, Ana Cortesão Costa, Catarina Teixeira, Mário Raimundo, Sónia Silva, Margarida Miranda, Edgar De Almeida

**Affiliations:** 1 Hospital Beatriz Ângelo, Departamento de Nefrologia, Loures, Portugal.; 2 Hospital Beatriz Ângelo, Departamento de Oftalmologia, Loures, Portugal.

**Keywords:** Atherosclerosis, Cholesterol, Embolism, Aterosclerose, Colesterol, Embolia

## Abstract

Atheroembolic renal disease (AERD) is a kidney manifestation of atherosclerosis as a systemic disease. AERD is defined as a renal impairment secondary to embolization of cholesterol crystals with consequent occlusion of renal vascularization. The current case report describes one patient with multiple risk factors but without any inciting event history who presents a very atypical clinical course of a severe and massive atheroembolic disease that developed spontaneously and silently.

## RESUMO

A doença renal ateroembólica (DRAE) é uma manifestação renal da aterosclerose enquanto patologia sistêmica. A DRAE é definida como uma disfunção renal secundária à embolização de cristais de colesterol seguida da oclusão da vascularização renal. O presente relato descreve o caso de um paciente com vários fatores de risco, porém sem um evento precipitante, que se apresentou com um curso clínico bastante atípico de doença ateroembólica grave de evolução espontânea e silenciosa.

## INTRODUCTION

Atheroembolic renal disease (AERD) is one of the multiple manifestations of atherosclerotic disease. The embolization of cholesterol crystals from atheromatous plaques is the lodging of cholesterol in smaller vessels of the kidneys, causing localized inflammation and ischemia. The condition typically affects the kidneys, skin, gastrointestinal tract, brain, and eyes.^1^

The release of cholesterol emboli into the circulation may occur spontaneously or, more frequently, after arterial manipulations, such as angioplasty, vascular surgery, stent placement, or use of anticoagulants and thrombolytic agents.^2^ Risk factors for AERD are the same as those for the development of atherosclerosis such as older age, male gender, diabetes, arterial hypertension, hypercholesterolemia, and smoking.^1^^,^ 3 The prognosis, both renal and vital, is poor.^2^^,^ 4 ^,^ 5

There is no specific therapeutic option for AERD, and limited unspecific care. Prevention measures, such as risk factors control and alternative approaches for arterial manipulation, may represent the best strategy.^1^^,^ 4

## CASE REPORT

A 70-year-old Caucasian man with long-term type 2 diabetes mellitus, arterial hypertension, dyslipidemia, and past smoking habits was admitted in the nephrology department with unspecific complaints of weakness and weight loss (5 kg in 3 months) associated with a rapidly progressive renal failure. His outpatient medications were metformin, simvastatin and enalapril. There was no history of new medications, surgical interventions, or other medical procedures. On admission, he was afebrile with a normal blood pressure. The physical examination was unremarkable.

Initial laboratory studies showed normocytic and normochromic anemia (Hb 10.5 g/dL), mild thrombocytopenia with normal lactate dehydrogenase, serum creatinine of 7.64 mg/dL (ten days before the value was 4 mg/dL), with normal anion-gap metabolic acidosis, C reactive protein of 4.1 mg/dL, and erythrocyte sedimentation rate (ESR) of 100 mm/h. Urine sediment had no alterations. Urinary protein/creatinine ratio was 297 mg/g. Laboratory studies performed 3 months before showed no anemia (Hb of 13g/dL) and a serum creatinine of 1.2 mg/dL.

Renal ultrasound revealed normal kidneys and no dilation of the urinary system. Chest X-ray was unremarkable. Based on this presentation a rapidly progressive glomerulonephritis was suspected. Considering the severity of renal impairment, empiric therapy with pulse methylprednisolone followed by oral prednisolone (1 mg/Kg/day) was initiated before the additional laboratory evaluation was available.

Complementary studies revealed hypertriglyceridemia (201 mg/dL) and hypercholesterolemia (total cholesterol of 211 mg/dL and LDL of 105 mg/dL). Viral serologies were negative, peripheral blood cultures were sterile, and complement levels (C3 and C4), antinuclear antibodies, serum electrophoresis, and anti-neutrophil cytoplasmic antibodies were normal.

**Figure 1:**
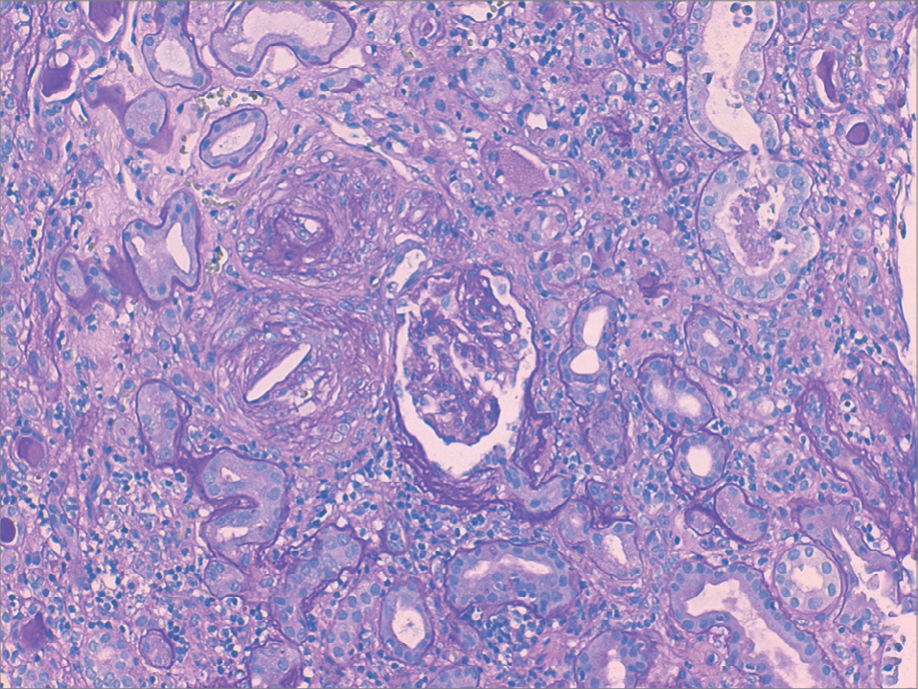
Periodic acid-Schiff staining shows slit-like cholesterol cleft within arterioles lumen, with cellular inflammatory reaction and lumen occlusion (100X).

**Figure 2:**
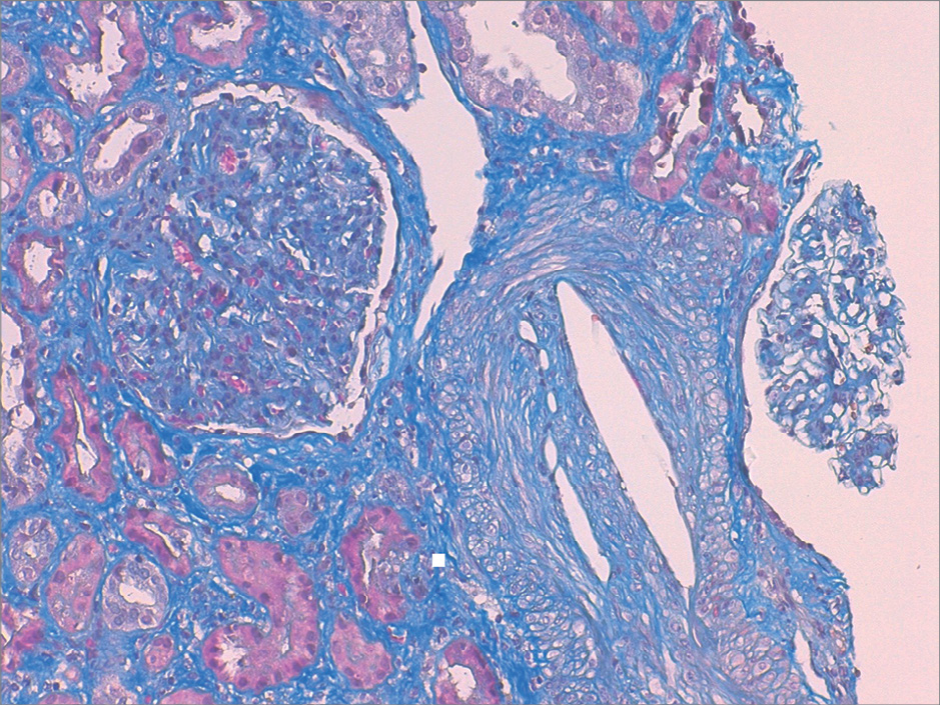
Masson trichrome stain show large cholesterol cleft within arteriole lumen, surrounded by interstitial fibrosis and tubular atrophy (200X).

Kidney biopsy was performed (Figure 1 and Figure 2). Light microscopy showed slit-like cholesterol clefts within arteries and arterioles lumen, with cellular inflammatory reaction and lumen occlusion. Glomerular basal membrane thickening and mesangial expansion was also present with interstitial fibrosis, lymphocytic infiltration, and tubular atrophy. Immunofluorescence revealed linear IgG deposit and albumin. Electron microscopy was not performed. These alterations were compatible with atheroembolic renal disease and diabetic nephropathy (stage IIb).

The patient showed a significant improvement with serum creatinine decreasing to 4.14 mg/dL at discharge. Prednisolone in tapering doses plus statin and antiagregation therapy with acetylsalicylic acid was prescribed.

**Figure 3:**
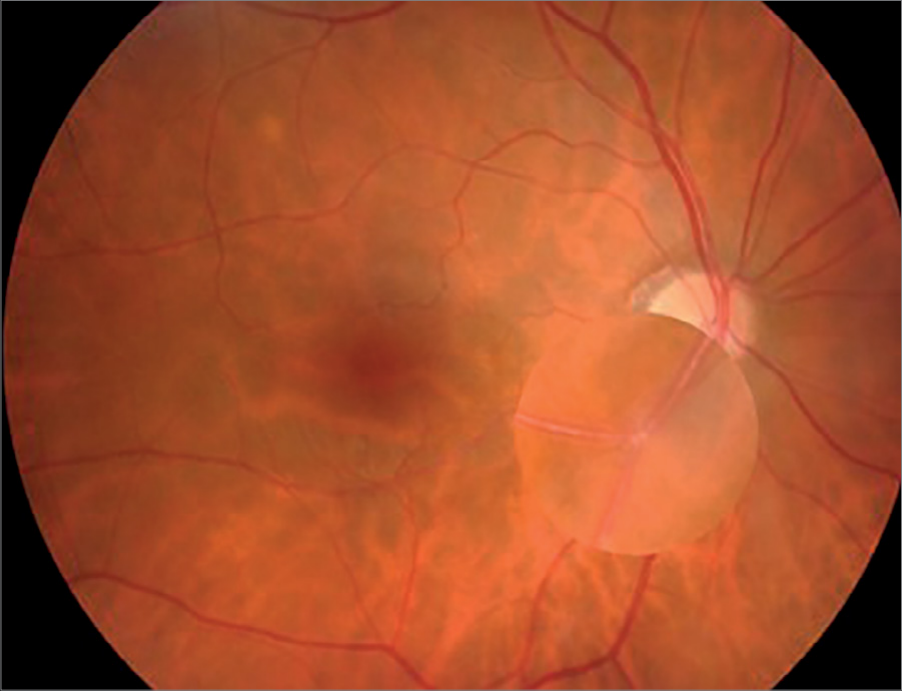
Retinography of the right eye highlighting an embolus in the lower temporal retinal artery.

Ten days later, he presented a sudden unilateral vision loss. Ophthalmological examination (Figure 3) revealed the presence of Hollenhorst plaques on retina and retinography confirmed the presence of retinal emboli. Clopidogrel was added to the previous therapeutic schema.

To better assess the extension of the vascular disease, a body tomography was performed, which revealed multiple vascular calcifications with irregular thrombosis in alternated aortic segments. Additionally, carotid ultrasound showed bilateral atherosclerotic disease, without major hemodynamic alterations.

Three months after the initial episode, the patient was asymptomatic, had a further improvement of renal function (serum creatinine of 1.4 mg/dL), and vision loss was also partially recovered.

## DISCUSSION

This patient presented with rapidly progressive renal failure of unclear etiology associated with anemia, increased ESR, and constitutional symptoms but classical signs of atheroembolic disease were absent. He had many risk factors for atherosclerotic disease but in the absence of arterial manipulation, anticoagulation, or fibrinolytic therapy that could trigger embolization, the diagnosis of AERD seemed unlikely.

Our patient had an atypical presentation of AERD because, despite the absence of a trigger event, he had a massive spontaneous embolization with severe and rapidly progressive renal insufficiency, opposite to a slowly progressive CKD, more commonly observed in spontaneous AERD. Additionally, at presentation, there was no sign of other organ involvement, but only unspecific complaints of weakness and weight loss. Only later, eye embolization was also evident. With such a severe form of kidney injury, a more severe and evident extra-renal disease would be expected at presentation, such as the presence poor arterial perfusion of the lower limbs.

Presence of diffuse aortic atherosclerosis is essential for development of AERD and the abdominal portion of the aorta is one of the main sources of emboli. (6) In our patient, a severe form of atherosclerosis was documented in the aorta in the CT scan. Findings of the renal biopsy demonstrated that renal injury was secondary to the occlusion of small caliber arteries by cholesterol crystals probably originated from ulcerated atherosclerotic plaques. These crystals also initiate a foreign body-type inflammatory response with endothelial proliferation and consequent fibrosis.^1^^,^ 5 ^,^ 7 Laboratory test findings in AERD are non-specific, such as anemia, thrombocytopenia, and elevation of inflammatory markers (leukocytosis, CPR, and ESR). Eosinophilia and low levels of C3 can also be documented.^1^^,^ 8 ^,^ 9 Urinalysis is usually benign as well as low-grade proteinuria. Eosinophiluria may be detected, and although highly suggestive of AERD, it is generally only present at early stages.^10^ Additional laboratory studies may be useful on detection of other organ lesions and allow ruling out alternative diagnosis such as small vessel vasculitis, occult infection or thrombotic microangiopathy.^11^

A classic triad has been described, which combines the exposure to an inciting event with acute/subacute renal injury and signs of peripheral atheroembolism such as skin involvement or the presence of cholesterol crystals in the retina (Hollenhorst plaques). In these cases, the diagnosis can be made clinically without the need for tissue analysis confirmation.^1^^,^ 5 ^,^ 12 In the present case, kidney biopsy was essential for the diagnosis because there was no other organ involved and no precipitating agent. The histological findings showed evidence of deposition of cholesterol crystals in the arterial vessels with a "negative" image in the form of a bi-convex lens corresponding to the place where the crystal was located, which is associated with perivascular inflammation.^1^^,^ 2 ^,^ 6

There is no specific treatment for AERD.^1^^,^ 2 ^,^ 10 Statins seem be beneficial in AERD, possibly due to its stabilizing role of atherosclerotic plaques through lipid control and anti-inflammatory effect.^5^ The use of anti-platelets therapy depends on the need of secondary prevention of cardiovascular disease. The use of anticoagulants and fibrinolytics should be avoided if possible in order to minimize the occurrence of new embolism.^3^^,^ 7 The use of steroids is very controversial, but their role in reducing the local inflammatory response might have been one of the mechanisms that led to the improvement of renal function in our patient.^5^^,^ 13 ^,^ 14 In this case, the decision to initiate steroids was made in the setting of a rapidly progressing renal insufficiency, before kidney biopsy was available. Nevertheless, the patient had a favorable response assuming a beneficial anti-inflammatory role of corticosteroids. Secondary prevention therapy, with statin and antiaggregation medications was also initiated. Three months later, the patient was asymptomatic with a significant improvement of renal function and visual acuity.

The authors reported an atypical presentation of a severe and massive atheroembolic disease that developed spontaneously. In patients with atherosclerosis disease, AERD should always be considered in the differential diagnosis of rapidly progressive renal insufficiency, despite unspecific symptoms or mild physical signs.
